# Considerations for Mitigation of the Psychological Impacts of COVID-19 in Older Adults

**DOI:** 10.30476/ijcbnm.2020.86362.1340

**Published:** 2020-07

**Authors:** Maryam Shaygan, Farzaneh Bahadori

**Affiliations:** 1 Community Based Psychiatric Care Research Center, Department of Nursing, School of Nursing and Midwifery, Shiraz University of Medical Sciences, Shiraz, Iran; 2 Department of Gerontology, University of Social Welfare & Rehabilitation Sciences, Tehran, Iran

**DEAR EDITOR**

There have been numerous outbreaks of infectious diseases in the world. On January 30, 2020, the World Health Organization (WHO) declared the outbreak of novel coronavirus disease (COVID-19) a Public Health Emergency. ^1^ The pandemic has caused not only the risk of death, but also too much psychological pressure on people all over the world. 

Older adults are more susceptible to severe infections, cascade of complications, disability, and death. ^2 , 3^ The elderly might also be more vulnerable to mental health problems because of their higher risk of infection. ^4^ They may experience adverse feelings such as fear of death because of the potential lethality of the illness. Some of them claim that dying from coronavirus is a horrible nuisance for them because none of their family members would be able to attend their funeral. They are also afraid of being hospitalized in medical facilities that lack sufficient equipment. They fear that if they contract the virus, no one will be able to visit or help them. The other fear the elderly experience is the infection of their spouses, children, and siblings. Dismal and disappointing news spreading via social media could also increase their fear and anxiety. Social distancing; reduced contact with others, especially loved ones; inability to take part in routine day-to-day activities (e.g. shopping for necessities); and cancellation of community events lead to boredom, frustration, and a sense of loneliness and isolation, which is distressing to older adults. This problem is particularly prominent in older adults who have limited access to the Internet-based services and smartphones. ^5 , 6^

The statement “coronavirus is mostly deadly to the elderly” increases a sense of fear and worthlessness in them. Some older adults have become extremely dependent on their children because they fear in-store shopping of essential items in large supermarkets, while they are not skilled in shopping online; this problem adds to their sense of worthlessness. 

Furthermore, restrictions on public transport and the fear of referring to treatment centers because of the contagion have become major barriers to receiving maintenance treatments for this age-group. Limited access to Internet-based services and smartphones increases the problems of this age-group. 

Therefore, stakeholders and health policymakers should take measures to prevent the potential mental health problems that might arise in older adults who are quarantined during the COVID-19 outbreak. Here are some suggestions to help mitigate the consequences of quarantine among older adults:

- Give older adults as much information as possible

Older adults often have catastrophic appraisals of any physical symptoms they experience, which may further increase feelings of fear and anxiety. This fear might be exacerbated by inadequate information. There is long-standing evidence to suggest that news has a direct impact on mental health. Feelings of uncertainty and doubt have long been associated with anxiety. ^6^ Therefore, it is necessary to ensure that older adults shape a good understanding of the disease and the reasons for quarantine. 

- Provide information to older adults mainly via broadcast media

Fast transmission of COVID-19 restricts face-to-face educational interventions. Moreover, Internet-based services, smartphones, social media (e.g. WhatsApp), and electronic books are not widely available to older adults. Therefore, governments and community-based health services should provide enough information about the disease via mass media, such as radio and television, which are most accessible to the elderly. 

- Provide community-based health services to address health issues of older adults

Community-based health services, including primary care, community nursing, and pharmacy services should provide telephone-based consultation services addressing health status, treatment, and medication management. Health services must also communicate adequate information to older adults in quarantine about what to do in the case of developing illness symptoms. It would help reassure the elderly that they would be taken care of if they fell ill.

- Provide mental health services to guide older people on how to manage their negative emotions and feelings of isolation

Mental health services should consider strategies to guide older people on how to manage stress, anger, and other negative emotions, and how to have effective communication with loved ones who do not live with them, so that the feelings of loneliness, stress, and anger are reduced. Such psychoeducational programs should be broadcast on mass media, which are most accessible to the elderly. Moreover, the elderly should receive practical advice on the ways which help them to reduce boredom and about the importance of having constant communication with their loved ones to overcome feelings of isolation. 

- Provide adequate supplies 

Public health authorities should provide the older adults with basic supplies, such as food, masks, and disinfectants during quarantine. Some studies have shown that insufficient basic supplies during quarantine are associated with the feelings of frustration, anxiety, and anger. ^6^

Considering the conditions in near future and the possibility of lengthy quarantines, respective organizations should practice effective strategies to enhance mental health in the society, especially among older adults. Some strategies that stakeholders and health policy-makers should implement to provide effective services to older adults who are quarantined at home during the COVID-19 pandemic are suggested in the present article.
